# An Oral Health Promotion Model Implemented in the Primorje-Gorski Kotar County

**DOI:** 10.3390/medicina61020217

**Published:** 2025-01-26

**Authors:** Danko Bakarčić, Nevenka Vlah, Odri Cicvarić, Dorotea Petrović, Marija Šimunović-Erpušina, Suzana Janković, Nataša Dragaš Zubalj, Sandro Kresina, Silvia Mohorić, Renata Gržić, Helena Glibotić Kresina, Nataša Ivančić Jokić

**Affiliations:** 1 Department of Pediatric Dentistry, Faculty of Dental Medicine, University of Rijeka, Clinical Hospital Center Rijeka, 51000 Rijeka, Croatia; danko.bakarcic@fdmri.uniri.hr (D.B.); odri.cicvaric@fdmri.uniri.hr (O.C.); dorotea.petrovic@fdmri.uniri.hr (D.P.); marija.simunovic.e@gmail.com (M.Š.-E.); natasa.ivancic.jokic@fdmri.uniri.hr (N.I.J.); 2 Department of Public Health, Teaching Institute of Public Health of Primorje-Gorski Kotar County, 51000 Rijeka, Croatia; nevenka.vlah@zzjzpgz.hr (N.V.); suzana.jankovic@zzjzpgz.hr (S.J.); natasa.dragas-zubalj@zzjzpgz.hr (N.D.Z.); sandro.kresina@zzjzpgz.hr (S.K.); silvia.mohoric@zzjzpgz.hr (S.M.); helena.glibotic-kresina@zzjzpgz.hr (H.G.K.); 3 Department of Prosthodontics, Faculty of Dental Medicine, University of Rijeka, Clinical Hospital Center Rijeka, 51000 Rijeka, Croatia

**Keywords:** child, dentistry, health promotion, oral health, preventive medicine

## Abstract

*Background and Objectives*: Global research has shown that 60–90% of school children have caries, and that oral health care is of great public health significance. We present the data of an oral health promotion Program conducted in the Primorje-Gorski Kotar County (PGC), Croatia, from 2008 to 2019. The Program includes comprehensive preventive oral status assessments of first- and fifth-grade elementary school students, as well as oral health promotional and preventative activities for preschool children, pregnant women, and new mothers. Here, we aimed to analyze the Program data and determine its applicability and sustainability. *Materials and Methods*: We assessed the changes in caries prevalence in first- and fifth-grade PGC students by comparing the 2008–2019 dental registry data on decayed, missing, and filled teeth for primary teeth (dmft)/decayed, missing, and filled teeth for permanent teeth (DMFT) index means. We also analyzed the data from the administrative Program reports. *Results*: We analyzed the dental registry data of 44,422 children in the PGC (21,714 first and 22,708 fifth grade). The average Program response rate was 83%. We noted a dmft/DMFT index decrease from 4.66 to 3.73 (first graders) and from 2.50 to 1.00 (fifth graders). The 2017–2019 dmft was significantly smaller than that of 2008–2009. There were 2336 workshops conducted in kindergartens, 1240 in first grades, and 1015 in fifth grades; health visitors educated 26,559 women. There was an increasing trend in the number of insured people under the age of six using pediatric oral health care. *Conclusions*: The Program improved the behavior and oral health of children and included various stakeholders, avoided additional financial expenses, increased the number of children in care, and proved its necessity and sustainability. It has been recognized on a national level and has led to the creation of two oral health care programs.

## 1. Introduction

In the modern world, children’s oral health has great societal and economic meaning. Regardless of the well-known nature of the disease and of its prevention methods, dental caries is currently the most prevalent civilization disease impacting most of the world’s population. Research shows that 60–90% of school children have caries; therefore, it represents a public health issue that requires serious consideration [1].

Each individual’s oral health depends on their oral hygiene habits, diet, economic status, and frequency of dental health care visits [2]. Caries prevention is based on measures and activities conducted in early childhood which, besides continuous fluoride application, high levels of oral hygiene, and adequate changes in diet and lifestyle, include systematic prevention and health education programs [3,4].

Research on the prevalence of childhood caries conducted in Croatia has been rare and sporadic. However, a trend of decayed, missing and filled teeth for permanent teeth (DMFT) index decline can be observed; in 12-year-old children, it was measured at 7 in 1968 and at 3.5 in 1999 [5,6]. Findings published in 2014 and 2015 showed a 4.8 DMFT index in fifth-grade students and 4.14 decayed, missing, and filled teeth for primary teeth (dmft) index and a 4.18 DMFT [7]. A study published in 2019 reported a 3.0 DMFT [3].

The lack of data and the decline of dental care quality in early childhood can be traced back to the events of the nineties, when, after the civil war and the creation of the independent Republic of Croatia, new laws and reforms took place. One such reform pertained to dental health care within the Healthcare Act. This reform, introduced in 1994, eliminated pediatric specialist practices from the national health system, which had previously conducted pediatric dentistry and prevention, including monitoring the dmft/DMFT indexes and other oral health indicators. Additionally, during this reform, the choice of the child’s dentist was handed over to the parents, when previously each kindergarten and school had an associated pediatric dentistry practice [8].

Recent locally conducted studies have shown that oral health in Croatia is still neglected and unsatisfactory, and that the awareness of the importance of oral health and its influence on general health is insufficient [9]. It is well-known that dental health needs to be addressed from the earliest age since children with healthy primary teeth have a very high likelihood of having healthy permanent dentition [10].

Guidelines state that pediatric oral health promotion begins even before birth, i.e., through the education of pregnant women on diet, microbe transmission, and oral health—both their own and that of their children. As first teeth appear in the first year of life, it is extremely important that parents are educated on proper diet and the need to delay and minimize the consumption of sugary foods, on the ways of infection transmission and caries development, and on methods of oral cavity cleaning and maintenance. Additionally, in the first year of life, it is important to have the first visit to the dentist; the best time for establishing proper dietary and hygiene habits is between the ages of one and four. From the age of five to ten, primary dentition is being replaced with permanent teeth. During this time, proper oral hygiene should be continued, along with the maintenance of a healthy diet, fluoridation, and more frequent dentist visits, ideally every six months [11].

Considering the guidelines above, it is clear that great strides can be made in maintaining oral health with minimal financial investments.

Despite positive examples being available both locally and in neighboring countries, the oral health status of children in Croatia has been neglected for many years, leading to a significant public health issue. Even though pediatric dental health care had previously existed, it was excluded during a health reform in the nineties; this in turn led to parents only taking their children to dental checkups when pain appeared. In order to change this and draw attention to the lack of oral health care, a local group of enthusiasts created an initiative which aimed to highlight the issue and demonstrate that prevention activities can be effective and later be implemented on a national level. Experts from the Teaching Institute of Public Health of Primorje-Gorski Kotar County (PGC), the Clinic for Dental Medicine of the Clinical Hospital Center Rijeka, and the Department of Pediatric Dentistry of the Faculty of Dental Medicine of the University of Rijeka have implemented the “Advancement of oral health in PGC children and youth” Program which is being conducted continuously since 2008 and has ensured the systematic oral health care of children and youth in the PGC. Similar programs have previously shown the usefulness of early detection of caries and oral health needs through the promotion of early prevention, as well as the importance of public health interventions [12,13,14,15,16,17].

The aim of this study was to analyze the program data and to assess the efficiency of this type of model in improving children’s oral health through evaluating the dmft/DMFT indexes of the target population, i.e., the students enrolling into first and fifth grades in the PGC from 2008 to 2019. Additionally, we aimed to assess whether there is a need for a similar program on a national level.

## 2. Materials and Methods

For the purposes of this research, we used the Program Dental Registry data collected by the Teaching Institute. The data were compiled from individual forms created according to the DMF Klein-Palmer system, on which clearly visible tooth surface cavities were noted as caries, and initial changes in transparency without cavitations were noted as healthy teeth [15]. To assess the response of first- and fifth-grade students to preventive dental exams and any concomitant dmft/DMFT index changes over the subsequent years, we analyzed the data of all the students enrolling into the first (average age: 6) and the fifth elementary school grades (average age: 12) from 2008 to 2019 whose oral health forms had been returned to the Teaching Institute, considering that these are the WHO recommended age groups for monitoring oral health [18].

The oral health data are collected at the Teaching Institute, in a dental database which enables precise monitoring of each tooth and holds accurate information regarding the tooth’s health, potential issues, and previous treatments. Additionally, the database facilitates administrative tasks by retaining basic patient information (name, sex, age, school, and dentist). Within the program, students enrolling in the first elementary grade were handed an oral health form at school which had to then be filled out by their chosen dentist. The filled-out form was given to their school medicine doctor as part of the health documentation for school enrollment. Fifth-grade students had their teeth examined at school by pediatric dentists. The exams were conducted in classrooms with artificial lighting and using single-use mirrors. The same forms as those for first-grade students were used and educational workshops on oral health were conducted. All forms were then returned to the Teaching Institute for data curation and analysis. Prior to the exams, the school provided the children with written consent forms for their parents. Each examined student received a notice for their parents outlining their oral status, the conducted workshops, and recommendations for oral health maintenance. During the school year, nursing graduates from the teams of School and Adolescent Medicine conducted one-hour school workshops for all present students on the importance of oral health maintenance and on adequate teeth brushing with demonstrations. Each student was also given a “For a healthy and pretty smile” brochure.

Since the program’s inception, health visitors have educated pregnant and postpartum women on the importance of oral health and handed them with “The health and smile of your children are in your hands” educational brochures.

Since 2014, the program has been implemented in PGC kindergartens as well; more intensive preventive oral health workshops were included alongside other daily activities. In kindergartens with adequate conditions, supervised teeth brushing was organized for children aged 3 to 6. After the workshops, the parents were given educational “Care for children’s teeth” brochures and the children were given educational coloring books. Additionally, upon kindergarten enrollment, the parents received instructions regarding the need to choose their child’s dentist.

We also analyzed all available program administrative data and reports, namely, the number of children assessed and educated by dentists within the program, the response rate, the number of educational workshops conducted in kindergartens, the number of children included in daily supervised teeth brushing, and the number of insured persons under the age of six. The response rate was calculated by dividing the number of children who returned their oral health forms by the number of all children enrolled into the first and fifth grades.

### Statistical Analysis

The data were analyzed in MedCalc, version 19.1.7 (MedCalc Software Ltd., Ostend, Belgium). The age of the included participants is shown as median and range (min-max). Categorical data are shown through absolute and relative frequencies. The differences between frequencies have been calculated with the Chi-square test, and as a post hoc analysis, the proportion *t*-test was used. The trend was calculated using the Chi-square test for trend.

Variance analysis (ANOVA) was used to determine the difference between dmft/DMFT indexes by age and sex, whereas the Scheffé test was used as a post hoc analysis. The significance level was set at *p* < 0.05 for all analyses.

The use of deidentified patient registry data for the purposes of this research was requested from the Teaching Institute. The research was approved by the Ethics Committee of the Teaching Institute of Public Health of the PGC (approval number: 07-700/147-23, 3 November 2023), and was conducted in accordance with the tenets of the 2008 Declaration of Helsinki and its 2013 amendment.

## 3. Results

From 2008 to 2019, 53,667 children were enrolled into the first and fifth grades: 27,323 into the first grade, and 26,344 into the fifth. Of them, 44,422 were examined; 21,714 first-grade students, median age 6 (range: 5–9) and 22,708 fifth-grade students, median age 12 (range: 10–15). The response rate was 86.2% for first and 79.4 % for fifth graders, and 82.8% for both combined. In the first grade, 10,567 boys and 10,046 girls (20,631 in total) and in the fifth, 10,885 boys and 10,112 girls (20,997 in total) were examined. In both grades combined, there were 21,452 boys (51.5%) and 20,176 (48.5%) girls; there was no statistically significant difference between the two (*p* = 0.204). Due to incomplete sex data, the data presented above were used in analyses that considered sex.

The program is currently being conducted in 90 kindergartens in the PGC area; 98% of kindergartens have adequate conditions and conduct teeth brushing once a day. During the observed period, 2336 workshops were conducted, and included 30,496 preschool children.

From 2008 to 2019, 1240 program workshops were conducted in the first grades and 21,714 students were assessed by dentists, and 1015 workshops were conducted with 22,708 students assessed in the fifth grades; in the same time period, health visitors educated 26,559 women.

Table 1 shows the measures of central tendency for the dmft index of first graders by years and the significant dmft differences by research years. The trend test showed that there was a statistically significant difference between groups in a certain direction (χ = 334.15, *p* < 0.001), i.e., that the dmft index diminished over time. Using the variance analysis, it was found that there was a significant difference in the dmft indexes (F[11, 21,702] = 9.75, *p* < 0.001) and by using the post hoc Scheffé test it was found that the dmft index was greater in the first program years than in the final years, as shown in Table 1.

The means, standard deviations, and ranges of the DMFT indexes of fifth-grade students are shown in Table 2.

The means, standard deviations, and ranges of the DMFT indexes of fifth-grade students are shown in Table 2.

The differences between the dmft indexes of first-grade students and the DMFT indexes of the fifth-grade students were also calculated and are shown in Table 3. The arithmetic mean of the dmft of first graders was 3.86 (standard deviation [SD] = 3.99) and that of the fifth-grade DMFT was 1.36 (SD = 1.70). The *t*-test showed that the first-grade students had a significantly higher dmft than the fifth grade DMFT (*p* < 0.01).

The oral health indicator for first-grade children, the dmft, declined from 4.66 to 3.73 and that for fifth-grade students, DMFT, from 2.50 to 1.00. With the two-way ANOVA analysis, we found a statistically significant difference by sex, with girls having a lower dmft (F[3, 41,624] = 5502, *p* < 0.001) and a lower DMFT (F[3, 41,624] = 5805, *p* < 0.001) than boys.

We found an increasing trend in the number of insured people using pediatric dental health care since birth until six years of age (χ = 137.27; *p* < 0.001) (Table 4), despite the continuous drop in birthrate (Table 4) [19].

From the total number of kindergarten-age preschool children (3–6 years old) in the PGC (N = 5693 children) who were included in the program, we found that 50% brushed their teeth daily.

## 4. Discussion

According to the 2008–2019 program data, in the PGC first-grade students the dmft index decreased from 4.66 to 3.73, whereas the DMFT index in fifth-grade students decreased from 2.50 to 1.00. From 2011 to 2015, the dmft index in first graders was continuously within the 4.17–4.23 range. With the program expansion in 2014 and the commencement of continuous, comprehensive, and systematic preventive activities in preschool institutions, an improvement in oral health indicators can be seen in first graders—the dmft was 4.0 in 2016, 3.8 in 2017 and 2018, and 3.7 in 2017. With this, we have shown that prevention programs that are intensely conducted in preschool establishments and are based on teeth brushing monitoring have a positive impact on the oral health of elementary school children, as seen in previous studies [13,15]. Additionally, the obtained findings show a DMFT reduction, i.e., an improvement in oral health status in fifth graders as well; as such, we can compare ourselves to other countries with a low DMFT index, namely Italy (1.1), Spain (1.07), Sweden (1.1), and Denmark and Switzerland (0.9) [20]. 

The dmft/DMFT decreases can be attributed to the main and additional activities of the program, namely, the preventive checkups, higher oral preventive awareness and more common prevention advice given by pediatricians and health visitors, earlier first dental visits, supervised teeth brushing in kindergartens, and preventive workshops.

Our study had several limitations. Firstly, the program inclusion was voluntary, therefore, we could only assess the dmft/DMFT indexes of children whose parents returned the filled-out forms to the Institute. Secondly, as there were no comparable programs that have been conducted locally, we were unable to compare our findings to those of our neighboring counties or other countries. Thirdly, we were unable to measure the influence of supervised teeth-brushing and the number of parents and children advised by pediatricians and health visitors. Aside from the program activities, it is possible that the dmft/DMFT index decrease was also influenced by various media and social network influences; however, this was not assessed in this study. Such a decrease would also be expected if the availability of sugary foods was decreased; however, no national policies have been implemented regarding sugar contents in food nor have there been any restrictions regarding what is available to children. Additionally, there has been no new evidence regarding positive changes in children’s overall diets.

The findings of a 2022 meta-analysis showed that there have been few studies on oral hygiene education worldwide; from the 18 in total, 2 have been conducted in Europe and only 3 have been conducted according to the WHO-outlined concept, which gives additional significance to the quality of our program considering the findings we obtained [14]. Aside from working with children, the program also motivates the parents to choose a dentist as soon as possible. Seeing that Croatia does not have organized oral health care for children, this responsibility is left to the parents, who oftentimes do not choose a dentist until caries-related issues manifest (pain, abscesses, etc.). The program ensured that most children enrolling in the first grade have a chosen dentist, which was not the case prior. By choosing a dentist, the child is included in the dental health care system, thus, changing the practice of dentists only being visited in dire need and imposing the option of preventive exams. The program model of having dentists visit schools has proven beneficial, similar to several studies which have shown that children included in school dental care programs are more motivated to visit their dentist and are more regular in their checkups and dental health care procedures [15,17,21].

The number of pediatric preventive dental exams has been increasing since the program’s inception, especially after the inclusion of kindergarten children in 2014. The prevention activities have also increased the parents’ awareness of the need to visit the dentist early, which can be seen in the increasing trend of insured people under the age of six. In the conduction of the program, health visitors were also included, educating pregnant and postpartum women on the importance of oral health maintenance, as they are especially motivated to maintain their children’s health. Being that in the early program years the DMFT index gradually declined and the dmft remained the same, it was necessary to commence more intensive prevention activities aimed at preschool children. Therefore, the program was expanded in 2014 by conducting workshops more intensively with kindergarteners and involving pediatricians in 2019 to cover all preschool children. The aim was that pediatricians, by following international, European, and Croatian guidelines, would send the children on their first dental checkup between 6–12 months of age, to ensure proper hygiene and feeding from the earliest age [22]. The program showed an improvement in children’s oral health through a reduction of the dmft and DMFT indexes. A further decrease at a similar rate is expected in the future, with a predicted variation during the COVID-19 pandemic period.

Various stakeholders have been important in the conduction of this program, each acting directly or indirectly within their domain for a common goal. Through their mobilization and interconnection, additional financial strains on the system can be avoided [23].

The sustainability of this model is demonstrated by the fact that it has been in place for over 15 years and has yielded measurable results; it has also proven easily modifiable. Dental health care has been made available through a proactive approach which encompasses children from before birth to age 18. By forming local clinics, organizing educational activities, systematically involving the parents, and monitoring and adjusting the program according to the response rate, free dental health care has been provided. The program has also achieved the aim of influencing national policies—it has been the model for the creation of two national oral health care programs: in 2017, the “Dental Passport” [24] program for schools, and in 2018 the “National Standards for Supervised Toothbrushing in Kindergartens and Primary Schools” [25] program.

The subsequent program development and research will focus on more detailed data monitoring as well as on data comparison with similar local and national programs.

## 5. Conclusions

Considering the possibilities of caries prevention and the advantages of early caries lesion diagnostics, it is necessary to establish preventive activities in kindergartens and schools.

Conducting these types of programs has great importance in encouraging children and parents to visit their dentists. Education on adequate oral hygiene, dietary habits, and oral disease prevention enables children to develop lifelong habits of maintaining their oral health. Here, by showing a reduction of the dmft/DMFT indexes on a local level, we have demonstrated that this type of program needs to be implemented at a national level and that there is an urgent need for a comprehensive national pediatric oral healthcare strategy.

## Figures and Tables

**Table 1:** Mean and standard deviations of first-grade students’ dmft from 2008 to 2019 and years with statistically significant differences.

dmft						
Year	N	Arithmetic Mean	SD	Min	Max	Statistically Significant dmft Difference: (*p* < 0.05)
2008	1822	4.66	4.19	0	20	2016–2019
2009	2033	4.47	4.24	0	20	2017–2019
2010	1912	4.29	4.01	0	20	**-**
2011	990	4.23	4.06	0	20	**-**
2012	945	4.08	4.06	0	20	**-**
2013	882	4.20	4.20	0	20	**-**
2014	2231	4.36	4.13	0	20	2017; 2019
2015	2331	4.17	4	0	18	
2016	2232	3.99	3.94	0	16	2010.
2017	2172	3.80	3.96	0	20	2008; 2009; 2014
2018	2134	3.83	3.92	0	20	2008–2009
2019	2030	3.73	3.81	0	20	2008; 2009; 2014
Total	21.714	3.86	3.99	0	20	

Abbreviations: dmft, decayed, missing, and filled teeth for primary teeth; SD, standard deviation; min, minimum; max, maximum.

**Table 2:** DMFT means and standard deviations in fifth-grade students from 2008 to 2019.

DMFT					
Year	N	Arithmetic Mean	SD	Min	Max
2008	122	2.50	2.21	0	10
2009	1369	2.00	2.14	0	18
2010	1238	1.31	1.60	0	10
2011	2475	1.64	1.84	0	13
2012	2195	1.44	1.77	0	12
2013	2610	1.34	1.67	0	12
2014	2250	1.32	1.58	0	22
2015	2014	1.41	1.68	0	9
2016	2181	1.42	1.74	0	24
2017	2065	0.95	1.33	0	6
2018	1968	1.20	1.60	0	10
2019	2221	1.01	1.50	0	12
Total	22,708	1.36	1.69	0	22

Abbreviations: DMFT, decayed, missing, and filled teeth for permanent teeth; SD, standard deviation; min, minimum; max, maximum.

**Table 3:** Differences in the dmft index in first-grade students and the DMFT index in fifth-grade students (2008–2019 combined).

	dmft	DMFT
Grade	1	5
N	21,714	22,708
Minimal value	0.000	0.000
Maximal value	20,000	22,000
Arithmetic mean	3.862	1.358
Median	3.000	1,000
95% CI	3.000 to 3.000	1.000 to 1.000
SD	3.9951	1.6945
25–75 P	0.000 to 7.000	0.000 to 2.000

Abbreviations: dmft, decayed, missing, and filled teeth for primary teeth; DMFT, decayed, missing, and filled teeth for permanent teeth; CI, confidence interval; SD, standard deviation; P, percentile.

**Table 4:** Frequency of insured people under the age of six that used dental health services each year (N; %).

Year	Number of Insured People 0–6	Number of People 0–6 Who Used Oral Health Care Services (N; %)		Number of Dental Examinations 0–6 (N; % in of Those Who Used Oral Health Services)	
2008	6152	3890	63.2%	3512	90.2%
2009	5437	3888	71.5%	2556	65.7%
2010	5732	3914	68.3%	3413	87.2%
2011	6486	4117	63.5%	3100	75.3%
2012	6167	4409	71.5%	3531	80.1%
2013	6532	4811	73.7%	2740	57%
2014	6514	4971	76.3%	2089	42%
2015	6490	5046	77.8%	3657	72.5%
2016	7312	5726	78.3%	4923	86%
2017	6905	5317		4589	86.3%
2018	6627	5112	77.1%	4054	79.3%
2019	6565	4559	69.4%	4322	94.8%
χ	137.27	*p* < 0.001			

## Data Availability

The original contributions presented in this study are included in the article. Further inquiries can be directed to the corresponding authors.
